# Sorafenib plus hepatic arterial infusion chemotherapy with cisplatin versus sorafenib for advanced hepatocellular carcinoma: randomized phase II trial

**DOI:** 10.1093/annonc/mdw323

**Published:** 2016-08-29

**Authors:** M. Ikeda, S. Shimizu, T. Sato, M. Morimoto, Y. Kojima, Y. Inaba, A. Hagihara, M. Kudo, S. Nakamori, S. Kaneko, R. Sugimoto, T. Tahara, T. Ohmura, K. Yasui, K. Sato, H. Ishii, J. Furuse, T. Okusaka

**Affiliations:** 1 Department of Hepatobiliary and Pancreatic Oncology, National Cancer Center Hospital East, Kashiwa; 2 Department of Biostatistics, Kyoto University School of Public Health, Kyoto; 3 Department of Hepatobiliary and Pancreatic Medical Oncology, Kanagawa Cancer Center, Yokohama; 4 Department of Gastroenterology, National Center for Global Health and Medicine Center Hospital, Tokyo; 5 Department of Diagnostic and Interventional Radiology, Aichi Cancer Center Hospital, Nagoya; 6 Department of Hepatology, Osaka City University Hospital, Osaka; 7 Department of Gastroenterology and Hepatology, Kinki University School of Medicine, Osaka; 8 Department of Hepatobiliary and Pancreatic Surgery, Osaka National Hospital, Osaka; 9 Department of Gastroenterology, Kanazawa University Hospital, Kanazawa; 10 Department of Hepato-Biliary-Pancreatology, National Hospital Organization Kyushu Cancer Center, Fukuoka; 11 Department of Gastroenterology, Saiseikai Utsunomiya Hospital, Tochigi; 12 Department of Gastroenterology, Sapporo Kosei General Hospital, Sapporo; 13 Department of Molecular Gastroenterology and Hepatology, Kyoto Prefectural University of Medicine, Kyoto; 14 Institute for Advancement of Clinical and Translational Science, Kyoto University Hospital, Kyoto; 15 Clinical Research Center, Shikoku Cancer Center, Matsuyama; 16 Department of Medical Oncology, Kyorin University, Tokyo; 17 Department of Hepatobiliary and Pancreatic Oncology, National Cancer Center Hospital, Tokyo, Japan

**Keywords:** cisplatin, hepatic arterial infusion chemotherapy, hepatocellular carcinoma, sorafenib, randomized phase II trial

## Abstract

**Background:**

Sorafenib (Sor) is acknowledged as a standard therapy for advanced hepatocellular carcinoma (HCC). This trial was conducted to evaluate the effect of addition of hepatic arterial infusion chemotherapy with cisplatin (SorCDDP) to Sor for the treatment of advanced HCC.

**Patients and methods:**

We conducted a multicenter open-labeled randomized phase II trial in chemo-naïve patients with advanced HCC with Child-Pugh scores of 5–7. Eligible patients were randomly assigned 2:1 to receive SorCDDP (sorafenib: 400 mg bid; cisplatin: 65 mg/m^2^, day 1, every 4–6 weeks) or Sor (400 mg bid). The primary end point was overall survival.

**Results:**

A total of 108 patients were randomized (Sor, *n* = 42; SorCDDP, *n* = 66). The median survival in the Sor and SorCDDP arms were 8.7 and 10.6 months, respectively [stratified hazard ratio (95% confidence interval), 0.60 (0.38–0.96), *P* = 0.031]. The median time to progression and the response rate were, respectively, 2.8 months and 7.3% in the Sor arm and 3.1 months and 21.7% in the SorCDDP arm. The adverse events were more frequent in the SorCDDP arm than in the Sor arm, but well-tolerated.

**Conclusion:**

SorCDDP yielded favorable overall survival when compared with Sor in patients with advanced HCC.

**Clinical Trial registration:**

UMIN-CTR (http://www.umin.ac.jp/ctr/index-j.htm), identification number: UMIN000005703.

## Abstract (precis)

In a randomized phase II study of sorafenib plus hepatic arterial infusion chemotherapy with cisplatin in comparison with sorafenib alone in patients with advanced hepatocellular carcinoma, it yielded favorable overall survival when compared with sorafenib alone. This is the first report of its effectiveness in relation to the overall survival in comparison with that of sorafenib alone in patients with advanced hepatocellular carcinoma.

## introduction

Sorafenib is currently acknowledged as a standard therapy for advanced hepatocellular carcinoma (HCC), and is available worldwide [1]. After the introduction of sorafenib, a number of phase III trials of various molecular-targeted agents versus sorafenib as first-line chemotherapy have been conducted, but none of the agents examined so far has shown superior survival benefit to sorafenib [1].

Hepatic arterial infusion chemotherapy (HAIC) is employed to treat patients with advanced HCC [2, 3]. This treatment modality is associated with increased local concentrations of the anticancer agents in the tumor and reduced systemic distribution of the drugs, and a stronger antitumor effect and lower incidence of systemic adverse reactions may be expected when compared with systemic chemotherapy. In fact, high response rates, favorable long-term outcomes, and acceptable toxicities with some chemotherapeutic regimens of HAIC have been reported [2, 3]. However, no consensus has been reached as to its place as a standard treatment of advanced HCC. Among HAI regimens, cisplatin alone can be easily administered using the Seldinger technique, without the need for an indwelling reservoir system [4]. In addition, sorafenib has been shown to interact with platinum transporter proteins [5], and to exert a synergistic anticancer effect with cisplatin in preclinical research [6]. Clinical trials of sorafenib used in combination with cisplatin have been carried out for various cancers [7–9], and favorable outcomes have been reported. Herein, we report the results of a randomized phase II trial of sorafenib plus HAIC with cisplatin (SorCDDP) versus sorafenib alone (Sor). The primary end point was the overall survival, while the secondary end points were the time to progression, response rate, and adverse events.

## methods-patients and methods

### patient eligibility

The patient inclusion criteria were as follows: advanced HCC confirmed histologically or by typical findings of hypervascular tumor on computed tomography (CT) or angiography and elevated serum alpha-fetoprotein (AFP), or protein induced by vitamin K absence or antagonist-II level; unsuitable for surgical resection, liver transplantation, local ablative therapy or transarterial chemoembolization (TACE); no prior history of chemotherapy; age 20–79 years old; presence of intrahepatic tumors affecting the prognosis irrespective of the presence of extrahepatic tumors; Eastern Cooperative Oncology Group Performance Status 0–1; adequate organ function [neutrophil count ≥1500 /mm^3^, hemoglobin ≥8.5 g/dl, platelet count ≥60 000 /mm^3^, serum total bilirubin ≤2.0 mg/dl, serum albumin ≥2.8 g/dl, aspartate aminotransferase (AST) and alanine aminotransferase (ALT) ≤5 times the upper limits of normal, serum creatinine ≤1.2 mg/dl, creatinine clearance ≥60 ml/min]; Child-Pugh score 5–7; HAIC technically feasible; written informed consent.

The main exclusion criteria were as follows: refractory pleural effusion or ascites; hepatic encephalopathy; severe and active co-morbidity or concomitant malignancy; allergic reaction to iodine contrast medium precluding angiography; pregnant and lactating females; females of childbearing age unless using effective contraception; and unsatisfactory general condition. Patients with hepatitis B or C virus infection were eligible for enrollment in this trial, provided they fulfilled the eligibility criterion pertaining to hepatic reserve.

### treatments

The enrolled patients were randomly assigned 2:1 to the SorCDDP arm or the Sor arm. Randomization was done centrally using a minimization method with biased-coin assignment [10]. The dynamic allocation factors were the presence of portal vein tumor thrombosis and extrahepatic metastasis. In patients of the SorCDDP arm, based on the results of a phase I trial [11], sorafenib (Nexavar®, Bayer Health Care Pharmaceuticals; West Haven, CT, USA) was administered orally at a dose of 400 mg bid, and cisplatin (IA call®, Nippon Kayaku Co., Ltd; Tokyo, Japan) was administered concurrently at 65 mg/m^2^/cycle via a catheter placed in the proper, right, or left hepatic artery, or another feeding artery, every 4–6 weeks. In patients of the Sor arm, sorafenib was administered orally at a dose of 400 mg bid. The sorafenib treatment in both arms was continued until tumor progression or unacceptable toxicity, and the HAIC with cisplatin was administered up to a maximum of six cycles until radiological or symptomatic tumor progression, unacceptable toxicity, or technical difficulty in repeating the HAIC. If the protocol therapies were discontinued, the patient was allowed to receive other anticancer treatment at the physician's discretion.

The occurrence of grade 4 hematological toxicity, grade 3 non-hematological toxicity was generally considered as indication for suspending the sorafenib administration. When the toxicities improved by at least one grade when compared with the suspension criteria, the treatment was resumed at a reduced dose of 400 mg daily. If additional dose reduction was required, the dose was reduced further to a single administration of 400 mg every other day.

The criteria for administering HAIC with cisplatin were as follows: neutrophil count ≥1200/mm^3^, platelet count ≥50 000/mm^3^, serum total bilirubin ≤3.0 mg/dl, serum AST or ALT levels ≤5 times the upper limit of normal, and a serum creatinine level ≤1.5 mg/dl. If the above parameters did not fall within the starting criteria, the HAIC with cisplatin was postponed until the criteria were fulfilled.

### response and toxicity assessment

Evaluation of the tumor response by dynamic CT or MRI was carried out every 6 weeks using the modified Response Evaluation Criteria in Solid Tumors (RECIST) [12]. The responses were evaluated centrally by three independent reviewers. Overall survival was measured from the date of enrollment to the date of death or the date of the last follow-up. Time to progression was defined as the time from the date of enrollment to the first documentation of disease progression or death. Assessment of adverse events was based on the National Cancer Institute Common Toxicity Criteria, version 4.0.

### statistical analysis

This was a multicenter open-labeled randomized phase II trial. The primary end point was overall survival stratified by the allocation factors, including the presence/absence of portal vein tumor thrombosis and extrahepatic metastases. If the median survival associated with Sor were assumed as 7.0 months and that of SorCDDP as 9.5 months, the hazard ratio (HR) was 0.74. SorCDDP would be judged as being favorable if the HR is 0.74 or lower. A total of 105 patients were needed to estimate the 1-year survival rate with an accuracy of ±10%. This study did not have sufficient statistical power to permit formal statistical comparison between the two arms.

The differences in the categorical data between the two groups were analyzed by Wilcoxon's test. The overall survival time and time to progression were estimated by using the Kaplan–Meier method and the curves were compared using the log-rank test. HRs of the treatment effects were estimated using a Cox regression model, and stratified results by dynamic allocation factors, including the presence/absence of portal vein tumor thrombosis and extrahepatic metastasis, as well as unstratified results, were presented. This clinical trial was conducted with the approval of the review board of each participating institution and in accordance with the Declaration of Helsinki. This trial is registered with UMIN-CTR (http://www.umin.ac.jp/ctr/index-j.htm), identification number (UMIN000005703). Patient registration, random treatment allocation, and data collection were managed by the Japan Clinical Research Support Unit data center. The integrity of the data was ensured through careful review by the staff of the data center, the coordinating investigators (MI and SS), and the trial statistician (TS). All the data were fixed on 28 December 2014, and all the analyses of efficacy were carried out based on the full analysis set (FAS) by the TS using SAS 9.4 and JMP Pro 11.

## results

### patient characteristics

**Figure 1.**
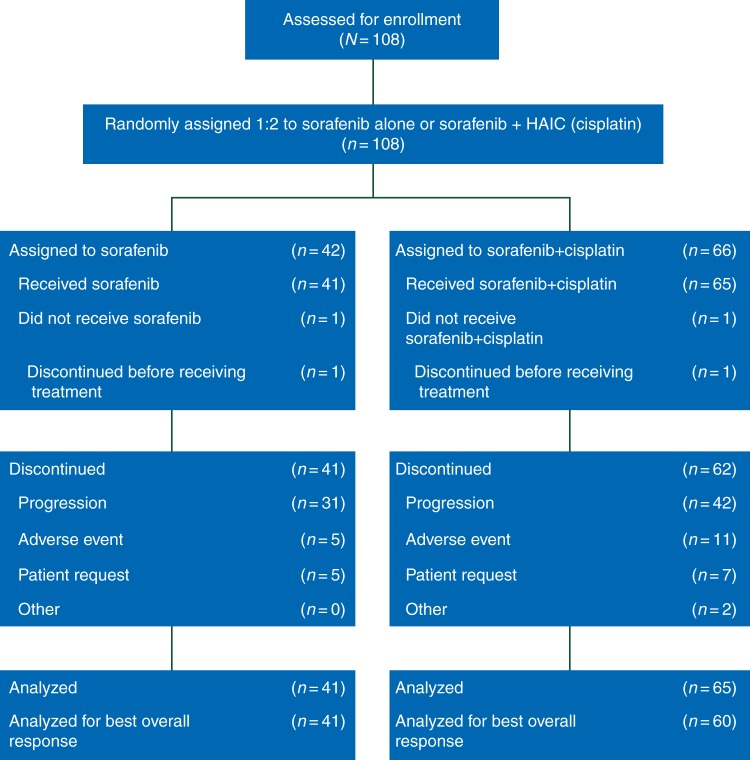
Consort diagram.

From June 2011 to December 2013, a total of 108 patients were enrolled and randomized into the two treatment arms (Figure 1). Forty-two patients were assigned to the Sor arm and 66 patients to the SorCDDP arm. While the planned random assignment was 2:1, the actual randomization ratio was 1.6:1, which was within random error. One patient from each of the arms could not receive the chemotherapy (development of paraplegia due to disease progression in one patient of the Sor arm, and withdrawal of informed consent in one patient of the SorCDDP arm). Therefore, the FAS included 41 patients in the Sor arm and 65 patients in the SorCDDP arm.

The patient characteristics of the 106 patients of the FAS are presented in Table 1. Seropositivity for hepatitis C viral antibody was more frequent in the Sor arm (*n* = 20, 48.8%) than in the SorCDDP arm (*n* = 18, 27.7%), and portal vein tumor thrombosis was less frequent in the Sor arm (*n* = 17, 41.5%) than in the SorCDDP arm (*n* = 40, 61.5%). In terms of all other variables, the patient characteristics were well-balanced.

**Table 1.** Baseline patient characteristics

Characteristics	Sorafenib alone (*n* = 41)		Sorafenib + HAIC (cisplatin) (*n* = 65)	
	Number of patients	%	Number of patients	%
Age, years				
Median	64		66	
Range	42–78		25–79	
Sex				
Male	32	78.1	56	86.2
Female	9	22.0	9	13.8
ECOG performance status				
0	33	80.5	50	76.9
1	8	19.5	15	23.1
Etiology				
Hepatitis B	9	22.0	22	33.8
Hepatitis C	20	48.8	18	27.7
Child-Pugh score				
5	27	65.9	38	58.5
6	12	29.3	19	29.2
7	2	4.9	8	12.3
Ascites	4	9.8	10	15.4
Previous therapy	21	51.2	33	50.8
Resection	6		17	
PEI/RFA	7		8	
TACE	14		23	
Radiation	1		1	
Other	2		1	
BCLC stage				
B	16	39.0	19	29.2
C	25	61.0	46	70.8
Portal vein tumor thrombosis	17	41.5	40	61.5
Vp1	0		4	10.0
Vp2	4	23.5	9	22.5
Vp3	7	41.4	14	35.0
Vp4	6	35.3	13	32.5
Extrahepatic spread	13	31.7	19	29.2
Lung	6		8	
Bone	3		1	
Lymph node	6		10	
Adrenal	1		1	
Other	2		4	
Number of tumors				
1	4	9.8	8	12.3
2	3	7.3	5	7.7
3	1	2.4	1	1.5
4	3	7.3	5	7.7
≥5	30	73.2	46	70.8
Maximum tumor size, cm				
Median	5.2		5.1	
Range	1.1–17.5		1.0–20.0	
Serum α-fetoprotein, ng/ml				
Median	188		223.5	
Range	2–749 412		1.2–394 944	
PIVKA II, mAU/ml				
Median	1790	1772		
Range	9–1 410,000		10–261 920	

ECOG, Eastern Cooperative Oncology Group; PEI/RFA, percutaneous ethanol injection/radiofrequency ablation; TACE, transarterial chemoembolization; BCLC, Barcelona Clinic Liver Cancer Group; Vp1, tumor thrombosis distal to the second branches of the portal vein; Vp2, tumor thrombosis in the second branches of the portal vein; Vp3, tumor thrombosis in the first branches of the portal vein; Vp4, tumor thrombosis in the main trunk of the portal vein or the opposite side branch of the portal vein; PIVKA II, protein induced by vitamin K absence or antagonist-II.

### treatments

By the data cutoff point, the protocol treatment had been discontinued in 41 patients of the Sor arm and 62 patients of the SorCDDP arm. The median number of cisplatin administrations and the median total dose of cisplatin in the SorCDDP arm were two times (range, 1–6 times) and 222 mg (range, 70–709 mg), respectively. The median dose intensity (range) was 488 mg/day (146–800 mg) in the Sor arm and 540 mg/day (193–800 mg) in the SorCDDP arm (*P* = 0.70). The proportion of patients in whom dose reduction of sorafenib was necessitated was 49.2% in the Sor arm and 63.4% in the SorCDDP arm. The median treatment duration (range) was 86 days (16–449 days) in the Sor arm and 75 days (4–881 days) in the SorCDDP arm (*P* = 0.58). After termination of the protocol treatment, 24 patients (59%) in the Sor arm and 40 patients (61.5%) in the SorCDDP arm received subsequent therapies, as follows: HAIC (8 and 19 patients, respectively), TACE (8 and 14 patients, respectively), local ablation (1 and 2 patients, respectively), other systemic chemotherapy (11 and 32 patients, respectively), palliative resection (2 and 5 patients, respectively), and radiotherapy (0 and 9 patients, respectively).

### efficacy

**Figure 2.**
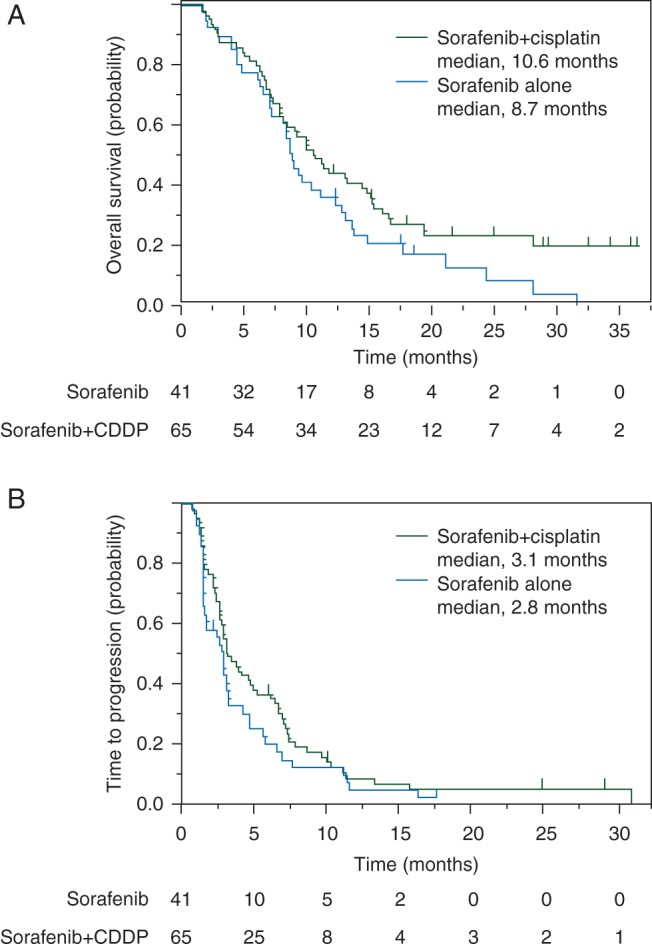
Kaplan–Meier curves of overall survival (A) and time to progression (B) in the sorafenib arm (blue line) and sorafenib plus hepatic arterial infusion chemotherapy with cisplatin arm (green line). The tick marks indicate censored cases.

**Figure 3.**
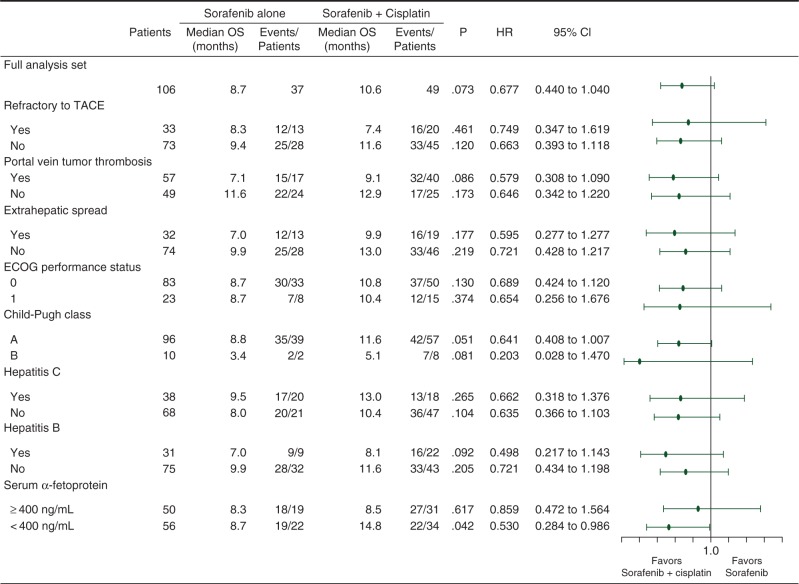
Forest plots showing subgroup analyses of the overall survival. TACE, transarterial chemoembolization; ECOG, Eastern Cooperative Oncology Group; OS, overall survival; HR, hazard ratio; CI, confidence interval.

At the final analysis, 37 patients of the Sor arm and 49 patients of the SorCDDP arm had died. The median survivals in the Sor and SorCDDP arms were 8.7 and 10.6 months, respectively (Figure 2A). The HR stratified by the allocation factors, including the presence/absence of portal vein tumor thrombosis and extrahepatic metastases (95% CI), was 0.60 (0.38–0.96), and *P*-value was 0.031. The crude HR [95% confidence interval (CI)] was 0.68 (0.44–1.049) (*P* = 0.073). The forest plot showing the pre-specified subgroup analyses of overall survival is shown in Figure 3. The patient subgroup with serum AFP <400 ng/ml showed a better overall survival in the SorCDDP arm (median 14.8 months) than in the Sor arm (median 8.7 months) (*P* = 0.042). At the data cutoff point, disease progression was observed in 39 patients in the Sor arm and 61 patients in the SorCDDP arm. The median time to progression was 2.8 months in the Sor arm and 3.1 months in the SorCDDP arm (Figure 2B). The crude HR was 0.78 (95% CI, 0.52–1.16, *P* = 0.212) and the HR stratified by the allocation factors was 0.78 (95% CI, 0.50–1.21, *P* = 0.257).

In the judgment by the central review, the number of patients evaluable by the modified RECIST criteria was 41 in the Sor arm and 60 patients in the SorCDDP arm. The response rate (95% CI) was 7.3% (1.5–19.9%) in the Sor arm and 21.7% (12.1–34.2%) in the SorCDDP arm (*P* = 0.09) (supplementary Figure 1, available at *Annals of Oncology* online).

### adverse events

The adverse events in both the arms during the entire treatment period until the final analysis are presented in Table 2. Neutropenia, leukocytopenia, decreased hemoglobin, thrombocytopenia, hyponatremia, nausea, and hiccups of all grades were more frequent in the SorCDDP arm than in the Sor arm. There were two treatment-related deaths in this series: one developed liver failure 9 months after the initiation of SorCDDP therapy, and the other developed pulmonary infection 2 months after the initiation of Sor therapy.

**Table 2.** Adverse events

	Sorafenib alone arm						Sorafenib + HAIC (cisplatin) arm					
	All grades		Grade 3		Grade 4		All grades		Grade 3		Grade 4	
	No. of pts	(%)	No. of pts	(%)	No. of pts	(%)	No. of pts	(%)	No. of pts	(%)	No. of pts	(%)
WBC decreased	18	43.9	0	0	0	0	49	75.4	12	18.5	0	0
Neu decreased	18	43.9	0	0	0	0	39	60	8	12.3	2	3.1
Hb decreased	30	73.2	2	4.9	1	2.4	58	89.2	6	9.2	2	3.1
Plt decreased	33	80.5	1	2.4	0	0	58	89.2	19	29.2	0	0
Bilirubin increased	29	70.7	5	12.2	0	0	48	73.8	6	9.2	2	3.1
AST increased	41	100	8	19.5	3	7.3	65	100	21	32.3	1	1.5
ALT increased	37	90.2	7	17.1	1	2.4	61	93.8	12	18.5	1	1.5
γGTP increased	37	90.2	14	34.1	2	4.9	63	96.9	21	32.3	3	4.6
Hypoalbuminemia	32	78	3	7.3	0	0	63	96.9	2	3.1	0	0
Cr increased	11	26.8	0	0	0	0	25	38.5	1	1.5	0	0
Hyponatremia	22	53.7	5	12.2	0	0	53	81.5	18	27.7	0	0
Amylase increased	21	52.5	0	0	0	0	41	64.1	10	15.6	1	1.6
Fatigue	16	39	1	2.4	–	–	28	43.1	7	10.8	–	–
Malaise	17	41.5	–	–	–	–	31	47.7	–	–	–	–
Appetite loss	22	53.7	1	2.4	0	0	45	69.2	4	6.2	2	3.1
Nausea	8	19.5	0	0	–	–	27	41.5	0	0	–	–
Vomiting	5	12.2	0	0	0	0	12	18.5	0	0	0	0
Diarrhea	17	41.5	1	2.4	0	0	23	35.4	4	6.2	0	0
Hand–foot synd	26	63.4	7	17.1	–	–	41	63.1	9	13.8	–	–
Skin rash	11	26.8	2	4.9	0	0	12	18.5	3	4.6	0	0
Hypertension	24	58.5	9	22	0	0	32	49.2	19	29.2	0	0
Hiccups	0	0	0	0	–	–	6	9.2	0	0	–	–

WBC, white blood count; Neu, neutrophils; Hb, hemoglobin; Plt, platelets; AST, aspartate aminotransferase; ALT, alanine aminotransferase; γGTP, γ-glutamyl transpeptidase; Cr, creatinine; synd, syndrome; pts, patients.

## discussion

In this study, SorCDDP yielded favorable overall survival when compared with Sor in patients with advanced HCC. The pre-specified HR stratified by the allocation factors (95% CI) was 0.60 (0.38–0.96), and *P*-value was 0.031. Because we had set the condition that SorCDDP would be judged as favorable if the HR for overall survival was 0.74 or lower, the primary end point of this study was met. In the pre-specified subgroup analysis of overall survival, the SorCDDP arm showed more favorable overall survival than the Sor arm in all the subgroups, and the efficacy of SorCDDP can be anticipated in almost all subjects who are suitable candidates for sorafenib treatment. In this trial, patients with hepatitis C viral infection showed a more favorable overall survival following sorafenib treatment than those with hepatitis B viral infection. However, it remains unknown whether patients with hepatitis C viral infection actually benefitted more from this treatment or not, because of the small sample size of this study. Furthermore, the overall survival in the SorCDDP arm was better than that in the Sor arm among the patients with serum AFP <400 ng/ml [crude HR, 0.53 (95% CI, 0.28–0.99)], whereas no difference was observed between the SorCDDP arm and the Sor arm among the patients with serum AFP ≥400 ng/ml. AFP may be one of the predictive biomarkers in patients receiving SorCDDP therapy, although the reason remains unknown.

Recently, immuno-oncology agents, such as tremelimumab [13] and nivolumab [14], have been introduced as promising agents for advanced HCC. The characteristics of these agents are a high response rate and long-lasting antitumor efficacy. In our study also, the response rate in the SorCDDP arm (21.7%) was threefold higher than that in the Sor arm (7.3%), and some patients in the SorCDDP arm showed long-lasting survival over 2 years. With regard to time to progression, the stratified HR by the allocation factors was 0.78 (95% CI, 0.59–1.21), and it was slightly worse than that of overall survival. In some phase III trials conducted for HCC, significant difference was observed in the time to progression or progression-free survival, but not in the overall survival [1]. Eventually, a negative result was concluded. However, in this study, the results were completely opposite. The most important difference between this study and these aforementioned trials may be in the anticancer treatments used: in this study, sorafenib was combined with a cytotoxic agent, while in the aforementioned phase III trials, it was used in combination with other molecular-targeted agents. Among patients showing marked tumor shrinkage on account of the favorable tumor shrinkage effect of SorCDDP, even a slight increase in the tumor size could result in their being classified as showing disease progression, whereas these patients may also show a prolonged overall survival because of the smaller tumor burden. This might also be the reason for the more favorable improvement of the overall survival than the time to progression.

The frequencies of the adverse events in the SorCDDP arm, except for those of neutropenia, leukocytopenia, hypohemoglobinemia, thrombocytopenia, hyponatremia, nausea and hiccups, were similar to those in the Sor arm. These adverse events were not severe. HAIC with cisplatin had only a mild toxicity profile [4] and the toxicities were not overlapped with the adverse effects of sorafenib. Therefore, SorCDDP therapy was also considered to be well-tolerated.

Intra-arterial administration of cisplatin was generally thought to be troublesome, requiring the insertion of a catheter into the tumor-feeding arteries. Recently, a phase III trial of sorafenib plus intra-arterial cisplatin and 5-fluorouracil versus sorafenib alone demonstrated no survival benefit [15]. One of the reasons could be the difficulty in placing the indwelling reservoir system. However, cisplatin is easily administered without the need for an indwelling reservoir system. Furthermore, this combined treatment is medico-economically very viable, because the additional cost of the angiographic procedure and cisplatin is approximately $2000 per session, which is less than the cost of the recently administered molecular-targeted agents or immuno-oncology agents.

In conclusion, this study demonstrated favorable overall survival in the SorCDDP arm when compared with that in the Sor arm in patients with advanced HCC, suggesting the effectiveness of HAIC against advanced HCC. However, since this study was only a randomized phase II trial, we could not arrive at any definitive conclusion with regard to the usefulness of sorafenib plus HAIC with cisplatin in the treatment of advanced HCC. A further phase III trial is being planned to confirm these results.

## funding

This work was supported in part by the National Cancer Center Research and Development Fund (23-A-22).

## disclosure

MI has received payment for lectures from Bayer Yakuhin and Nippon Kayaku; has been a consultant for Bayer Yakuhin; and has received research funding from Bayer Yakuhin. SS has received payment for lectures from Nippon Kayaku. YI has received payment for lectures from Bayer Yakuhin and Nippon Kayaku; and has received research funding from Bayer Yakuhin and Nippon Kayaku. AH has been on the speakers' bureau for Bayer Yakuhin. TO has received payment for lectures from Bayer Yakuhin. JF has received payment for lectures from Bayer Yakuhin and Nippon Kayaku; has been on advisory arrangements for Bayer Yakuhin; and received research funding from Bayer Yakuhin and Nippon Kayaku. TO has received research funding from Bayer Yakuhin and has received payment for lectures from Nippon Kayaku. All remaining authors have declared no conflicts of interest.

## Supplementary Material

Supplementary Data
