# Supplementary material for: Sorafenib plus hepatic arterial infusion chemotherapy with cisplatin versus sorafenib for advanced hepatocellular carcinoma: randomized phase II trial
Source: Ann Oncol. 2016 Aug 29;27(11):2090–6. doi: 10.1093/annonc/mdw323 (PMC5091321; doi:10.1093/annonc/mdw323)
Supplement: Supplementary Data [file supp_27_11_2090__index.html]

Sorafenib plus hepatic arterial infusion chemotherapy with cisplatin versus sorafenib for advanced hepatocellular carcinoma: randomized phase II trial — Sorafenib plus hepatic arterial infusion chemotherapy with cisplatin versus sorafenib for advanced hepatocellular carcinoma: randomized phase II trial — Supplementary Data 

# Sorafenib plus hepatic arterial infusion chemotherapy with cisplatin versus sorafenib for advanced hepatocellular carcinoma: randomized phase II trial

## Supplementary Data

Supplementary Data

- Supplementary Figure 1 - docx file
