# Supplementary material for: Sorafenib plus hepatic arterial infusion chemotherapy with cisplatin versus sorafenib for advanced hepatocellular carcinoma: randomized phase II trial
Source: Ann Oncol. 2016 Aug 29;27(11):2090–6. doi: 10.1093/annonc/mdw323 (PMC5091321; doi:10.1093/annonc/mdw323)

Supplemental figure 1. Waterfall plots of the sorafenib arm (A) and sorafenib plus hepatic arterial infusion chemotherapy with cisplatin arm (B)


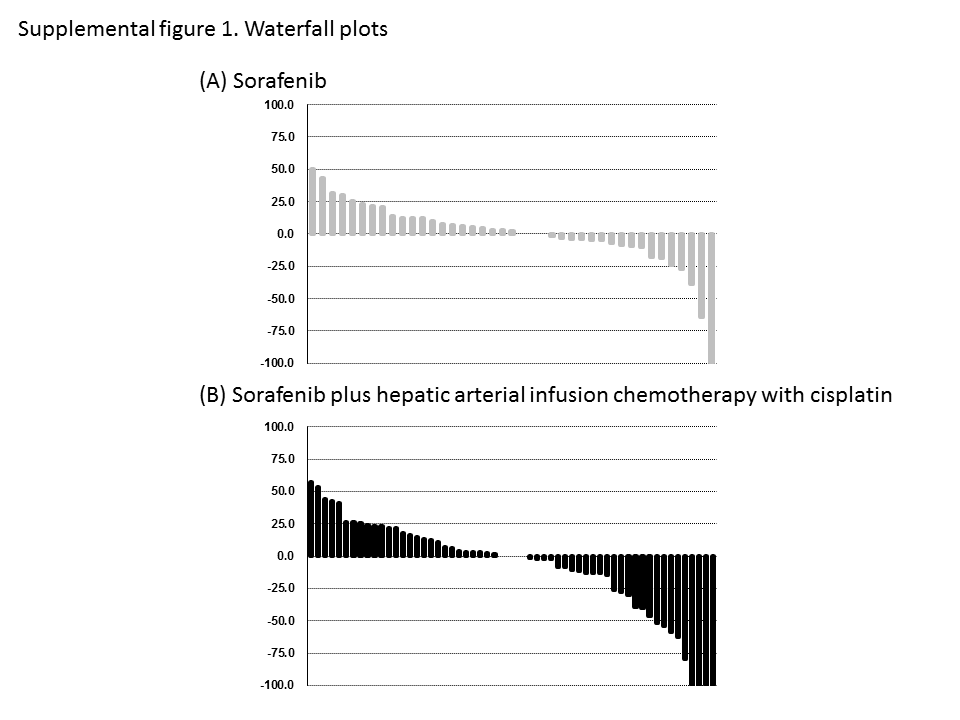

Supplement: Supplementary Data [file supp_mdw323_mdw323supp_fig1.docx]
